# Integration of proteomics and transcriptomics to construct a prognostic signature of renal clear cell carcinoma

**DOI:** 10.7150/ijms.99992

**Published:** 2024-08-19

**Authors:** Guangyang Cheng, Zhaokai Zhou, Shiqi Li, Zhuo Ye, Yan Wang, Jianguo Wen, Chuanchuan Ren

**Affiliations:** 1 Department of Urology, The First Affiliated Hospital of Zhengzhou University, Henan, Zhengzhou 450052, China.; 2 Henan Joint International Pediatric Urodynamic Laboratory, The First Affiliated Hospital of Zhengzhou University, Henan, Zhengzhou 450052, China.; 3 Bladder Structure and Function Reconstruction Henan Engineering Laboratory, The First Affiliated Hospital of Zhengzhou University, Henan, Zhengzhou 450052, China.

**Keywords:** Proteomics, Transcriptomics, Renal clear cell carcinoma, Prognostic signature, tumor microenvironment

## Abstract

**Background:** Protein information is often replaced by RNA data in studies to understand cancer-related biological processes or molecular functions, and proteins of prognostic significance in Kidney clear cell carcinoma (KIRC) remain to be mined.

**Methods:** The cancer genome atlas program (TCGA) data was utilized to screen for proteins that are prognostically significant in KIRC. Machine learning algorithms were employed to develop protein prognostic models. Additionally, immune infiltration abundance, somatic mutation differences, and immunotherapeutic responses were analyzed in various protein risk subgroups. Ultimately, the validation of protein-coding genes was confirmed by utilizing an online database and implementing quantitative real-time PCR (qRT-PCR).

**Results:** The patients were divided into two risk categories based on prognostic proteins, and notable disparities in both overall survival (OS) and progression free interval (PFI) were observed between the two groups. The OS was more unfavorable in the high-risk group, and there was a noteworthy disparity in the level of immune infiltration observed between the two groups. In addition, the nomogram showed high accuracy in predicting survival in KIRC patients.

**Conclusion:** In this research, we elucidated the core proteins associated with prognosis in terms of survival prediction, immunotherapeutic response, somatic mutation, and immune microenvironment. Additionally, we have developed a reliable prognostic model with excellent predictive capabilities.

## Introduction

Renal cancer is the most frequently occurring tumor of the urinary system^1^, and also has the highest mortality rate in urological tumors^2^. Around 85% of tumors in the kidneys are renal cell carcinomas, with about 70% being clear cell carcinomas of the kidney. Renal cell carcinoma (RCC) makes up around 3.8% of all newly diagnosed cancers, typically affecting individuals at a median age of 64 years^3^. However, those with distant metastases experience a significantly lower 5-year survival rate of 0% to 10%^4^. Despite the utilization of molecularly targeted treatments like inhibitors of tyrosine kinase and rapamycin for advanced renal cancer^5^, ^6^, finding a solution to enhance the OS and progression-free survival (PFS) of patients remains a Gordian knot. Constructing novel prognostic models could weigh better treatment modalities based on patient conditions to maximize clinical benefit and provide novel insights for precision medicine treatment decisions.

Proteins, as important mediators in biological processes and also responsible for the gene coding functions of cells, are demonstrated as a nexus with tumorigenesis and progression^7^. In most research, protein expression data are replaced by RNA sequencing data, but often with poor expected results^8^. To bridge the gap between genomic data and functional proteins, it is necessary to utilize proteomics and convert this information^9^. Recently, proteomic studies have enhanced insights into the biological basis and prognostic assessment of KIRC and revealed potential therapeutic targets^10^, ^11^. Therefore, further proteomic studies in KIRC, especially biomarker discovery, could provide novel prognostic molecular markers and effective treatment to win the war against KIRC.

For this research, we conducted transcriptomic and proteomic examinations from the TCGA-KIRC data. Comprehensive data analyses revealed different proteomic isoforms of KIRC, and a seven-protein-based prognostic model was constructed by linking the proteomic features of KIRC to clinical outcomes. In addition, we analyzed the relationship of protein prognostic models with immune infiltration and immune microenvironment. These results may provide a basis for predicting the outcome and therapeutic effects of KIRC.

## Materials and methods

### Data acquisition

The proteomic data, RNA-seq data, and clinical data of KIRC were downloaded from the genomic data commons (GDC) website. Missing values in the protein expression data were interpolated using the "impute" R package, and expression values for the same protein were averaged. For RNA-seq data, genes with overall low expression were removed and expression values for the same genes were averaged. For clinical data, patients with missing essential data were removed. The clinical characteristics of these samples are detailed in Supplementary Table S1.

### Screening and validation of model proteins

The "caret" R package was used to randomly divide 474 patients into training and test groups according to 5:5. Proteins that showed a significant correlation with prognosis were identified through COX regression analysis (*p*<0.01) in the training group. Subsequently, proteins were selected from the pool of prognosis-related proteins using least absolute shrinkage and selection operator (LASSO) regression. In the end, a multivariate COX regression model was built and the proteins for the final model were obtained through stepwise regression. Risk scores were calculated after screening the modeled proteins using the following formula: 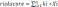, where k and 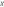 denote the relative expression level of the protein and the regression coefficient. The sample was divided into two risk groups using the median of the prognostic protein risk score as the cutoff value.

### Construction of nomogram

To facilitate the clinical application of prognostic models, we plotted nomogram using the "rms" and "regplot" R packages, along with calibration curves and receiver operating characteristic (ROC) curves.

### Consistent cluster analysis of prognostic proteins

The samples were typed according to the expression of prognostic proteins using the "ConsensusClusterPlus" R package, and the number of samples typed was determined according to the optimal *K*-value.

### Immune infiltration analysis, gene set enrichment analysis (GSEA), and tumor immune iysfunction and ixclusion (TIDE)

GSEA analysis was performed using the "clusterProfiler" R package, and GSEA analysis using the “c2.cp.kegg.symbols” gene set downloaded from the molecular signatures database (MSigDB) database. CIBERSORT was utilized to conduct immune infiltration analysis, and a boxplot illustrating the variations in immune cell infiltration among various subgroups was generated. Immunotherapy responses were analyzed using the TIDE website.

### Online database to validate expression of prognostic proteins and protein-coding genes

The human protein atlas (HPA) database was utilized to confirm the expression of prototype proteins in both normal and KIRC tissues. Additionally, the tumor immune single-cell hub (TISCH) database was employed to observe the expression of genes encoding proteins in various cell clusters. Furthermore, the cancer single-cell state atlas (CancerSEA) database was utilized to examine the relationship between the function of cancer cells and genes encoding proteins at the individual cell level. The BEST website was utilized to analyze the differential expression of genes.

### Cell culture

Human renal cortical proximal tubular epithelial cells (HK-2) and human renal cell adenocarcinoma cells (769-P, 786-O, and ACHN) were used for this study, both provided by Servicebio, Ltd (Wuhan, China). Among them, HK-2 and ACHN were cultured in MEM medium containing 10% fetal bovine serum (FBS) and 1% penicillin-streptomycin (P/S). 769-P and 786-O were cultured in RPMI 1640 medium containing 10% FBS and 1% P/S in a humidified incubator at 37°C with 5% CO_2_.

### RNA extraction and qRT-PCR

Total RNA was extracted from cells using RNAeasy™ Animal RNA Extraction Kit (RR0026, Beyotime, Shanghai, China). cDNA synthesis was performed using PrimeScript™ RT reagent Kit with gDNA Eraser (RR047A, Takara, Beijing, China), and real-time quantitative PCR was performed using TB Green® Premix Ex Taq™ II (RR820A, Takara, Beijing, China). qPCR primers were synthesized by Sangon Biotech, Ltd (Shanghai, China) (Table 1). β-actin was used as a standardized internal reference gene. The relative gene expression levels were calculated using the 2^-ΔΔCt^ method.

### Statistical analysis

The prognostic significance was evaluated through Kaplan-Meier (K-M) analysis and COX analysis, comparing groups using the Wilcoxon test for numerical variables and the chi-square test for categorical variables. R4.3.2 was utilized for statistical analyses, with significance defined as *p*<0.05 unless stated otherwise.

## Results

### KIRC prognosis-related protein screening

**Figure 1:**
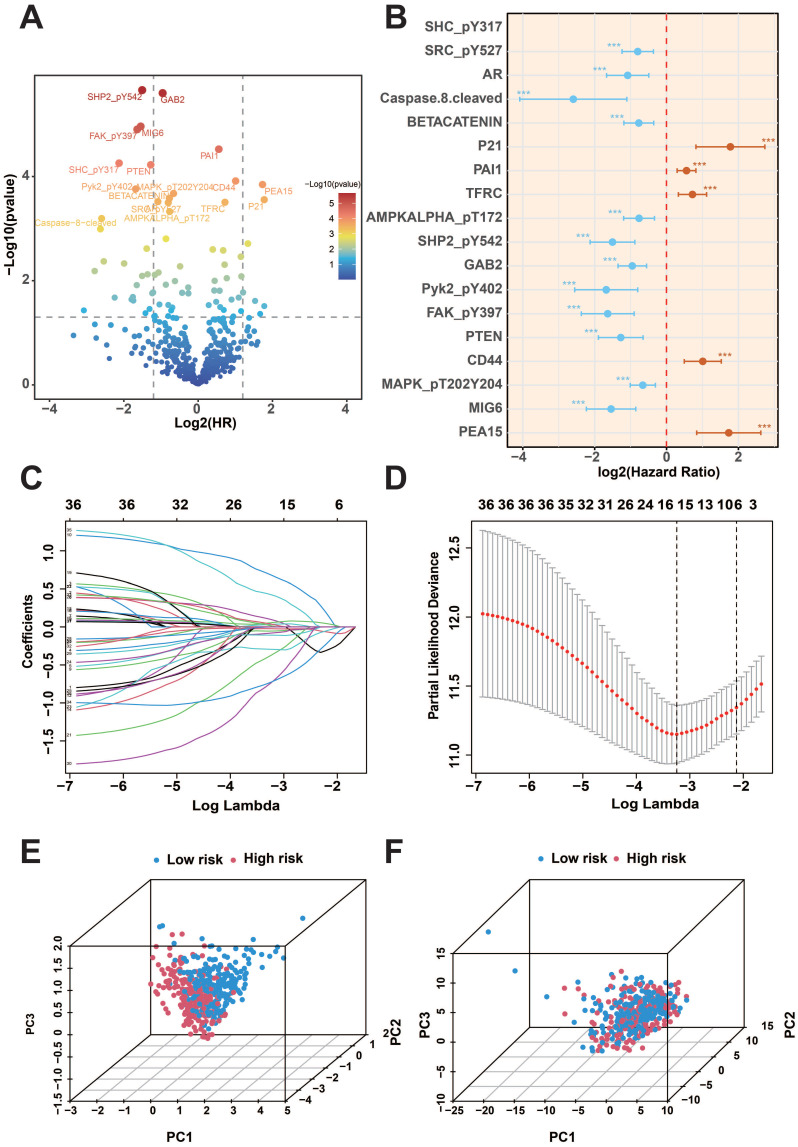
KIRC prognosis-related protein screening. A: COX regression analysis of protein prognostic volcano plots. B: Forest plot of prognostic-related proteins. C-D: Results of LASSO regression analysis and cross-validation. E-F: PCA analysis of risk scores. (***p<0.001).

Firstly, we analyzed the proteomics data of TCGA-KIRC, and after screening, we finally obtained 474 valid samples and 36 prognostic proteins that were significantly correlated out of survival (*p*<0.01) and visualized the results on volcano and forest plots (Figure 1A-B). Subsequently, the LASSO and COX regression model was constructed to further screen prognostic proteins, and the final model identified the best performance when 7 proteins (ACC1, P21, P70S6K_pT389, SMAC, BRAF_pS445, MIG6, PEA15) were identified (Figure 1C-D). Patients were categorized into two groups based on the calculated risk score for each patient using regression coefficients, with the median risk score serving as the cutoff value. Principal component analysis (PCA) found that risk score could cluster samples well (Figure 1E-F).

### Prognostic protein model validation

**Figure 2:**
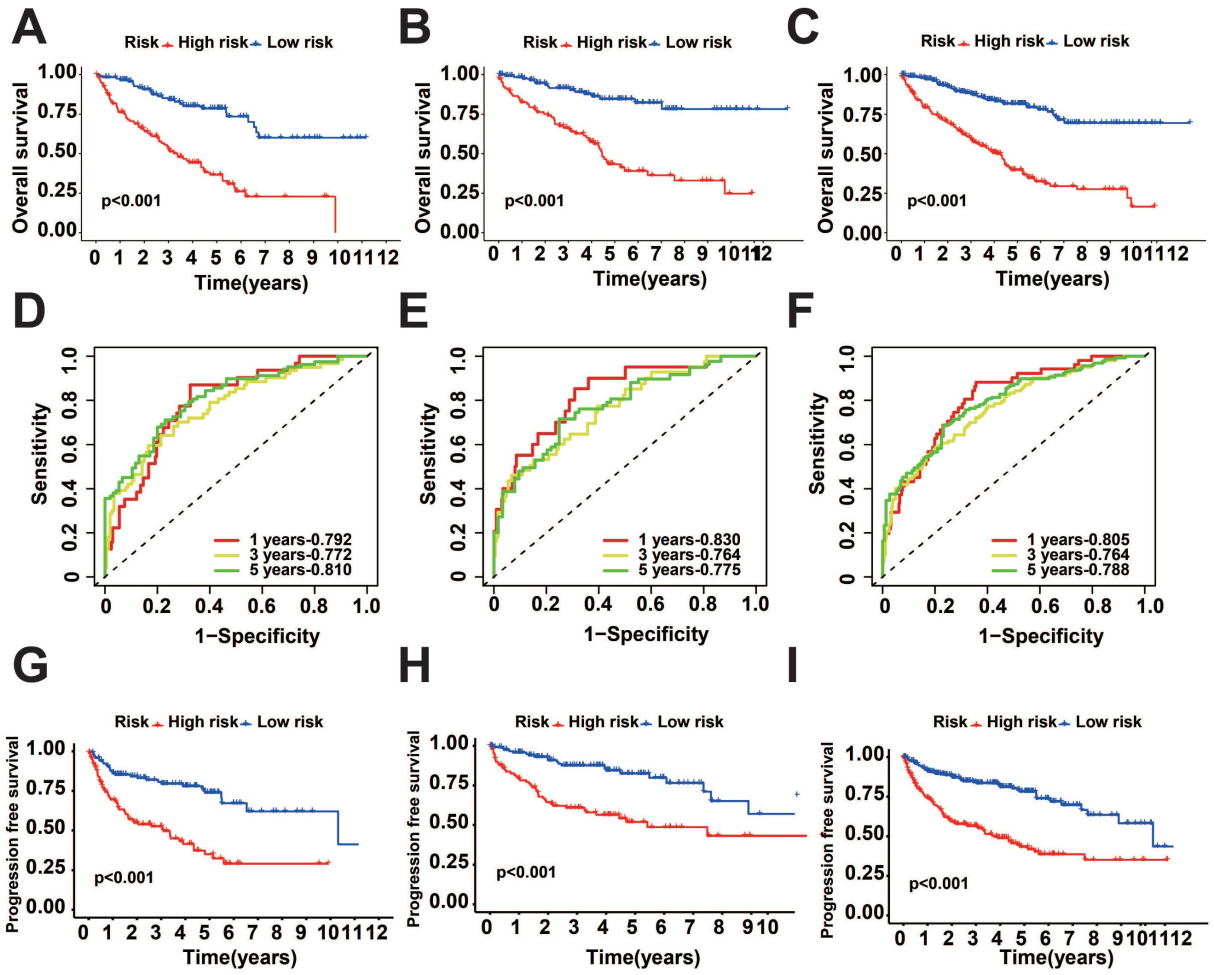
KIRC prognosis-related protein survival analysis. A-C: K-M survival curves for OS between the two groups in the training, test, and overall groups. D-F: ROC curves predicting OS in the training, test, and overall groups. G-I: K-M survival curves for PFI between the two groups in the training, test, and overall groups.

**Figure 3:**
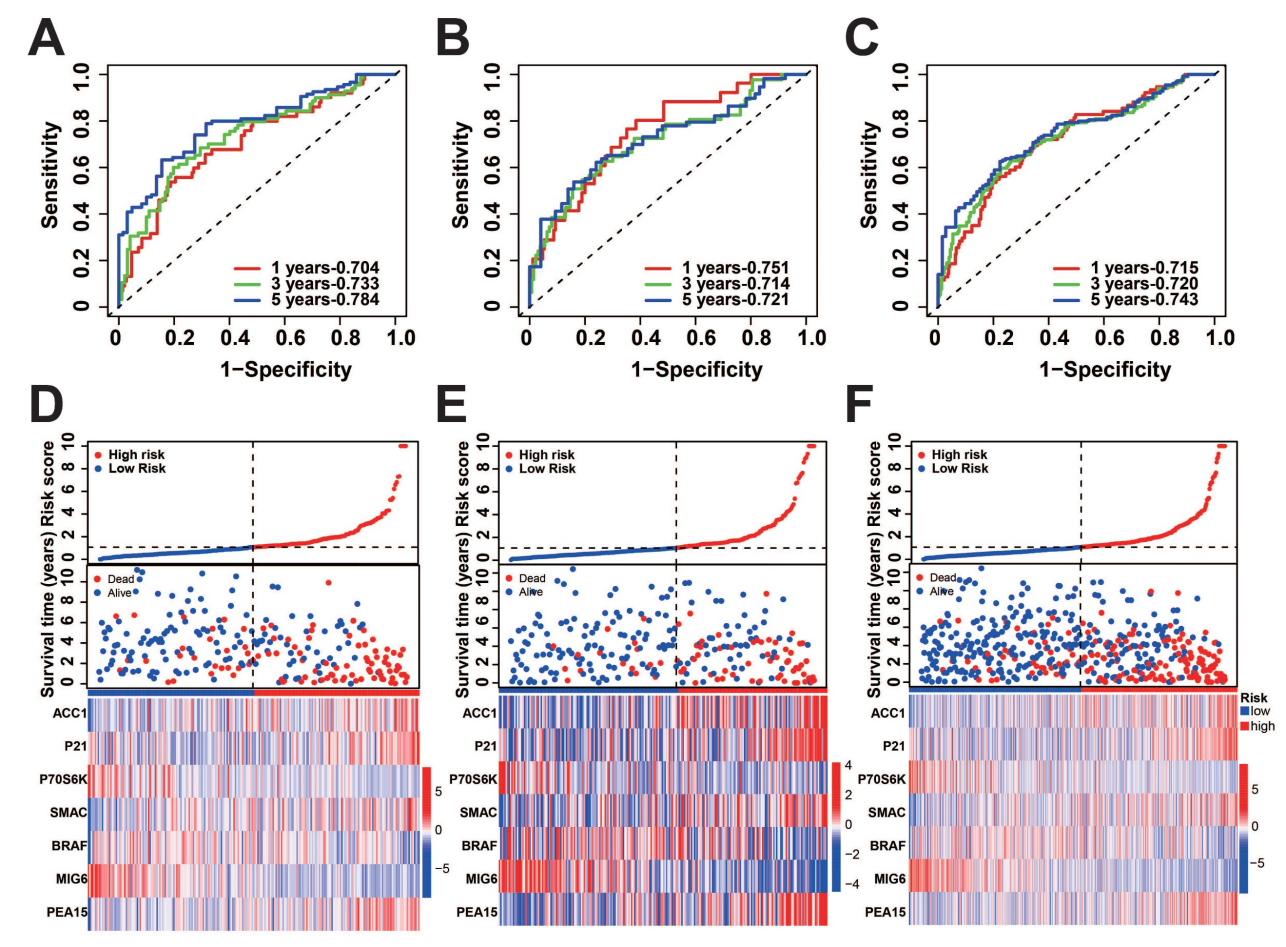
Distribution of risk score. A-C: Predicted ROC curves for PFI in the training, test, and overall groups. D-F: Distribution of risk scores across cohorts.

To verify the precision of the model, we also computed the risk scores for the validation group. Next, we conducted a survival analysis on the two groups and illustrated the disparities in survival using K-M curves. To assess the precision of the model, the corresponding ROC graphs were generated. In all cohorts, it was discovered that the high-risk score group had a considerably worse prognosis (Figure 2A-C, G-I). According to the ROC curves, the prognostic protein model exhibited a positive predictive performance for OS and PFI (OS-5 Year: Train Cohort: 0.810, Test Cohort: 0.775, TCGA whole Cohort: 0.788, PFI-5 Year: Train Cohort: 0.784, Test Cohort: 0.721, TCGA Whole Cohort: 0.743, Figure 2D-F, Figure 3A-C). Moreover, we plotted the sample distribution of risk scores for the three cohorts according to subgroups, and the expression of the modeled proteins is shown in the heatmap (Figure 3D-F). Finally, we examined the variations in survival rates among different risk subgroups based on clinical factors. The findings indicated that high-risk patients had unfavorable survival outcomes in subgroups categorized by age (≤ 65 and > 65), gender, tissue Grade levels, clinical stages, and TNM stages (*p*<0.001, Figure S1A-E). However, the survival differences were not statistically significant in the N1 stage (*p*=0.082, Figure S1F) and M1 stage (*p*=0.057, Figure S1G).

### Somatic mutation profile of tumor samples

**Figure 4:**
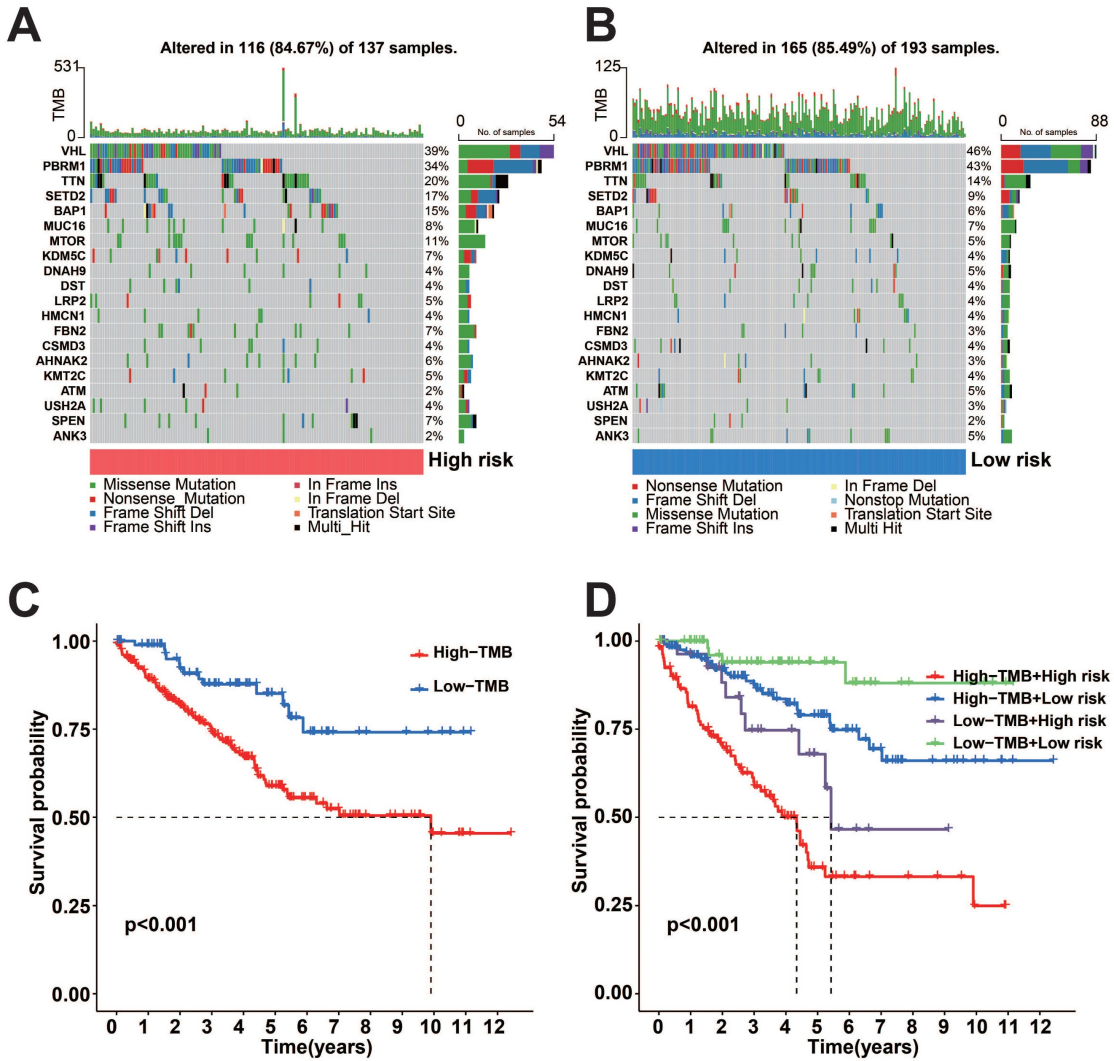
Somatic mutation analysis and survival analysis of model proteins in two risk subgroups. A-B: Gene mutations in the two risk subgroups. C: K-M survival curves for OS between high and low TMB groups. D: K-M survival curves for OS between the two TMB groups and the two risk groups.

To uncover genetic changes in the subgroups with high and low risks, and understand the connection between genetic mutations and patient survival, we conducted a waterfall plot analysis using copy number variation (CNV) data for TCGA-KIRC (Figure 4A-B). The findings indicated that both subgroups with high and low risk demonstrated elevated rates of mutation. After classifying patients based on the median tumor mutational load (TMB), we observed that the high TMB group exhibited a more unfavorable prognosis compared to the low TMB group (Figure 4C). The final combined survival analysis of TMB grouping and risk score grouping showed that the high TMB and high-risk score group had the most unfavorable prognosis (Figure 4D).

### Clinical correlation of prognostic proteins

**Figure 5:**
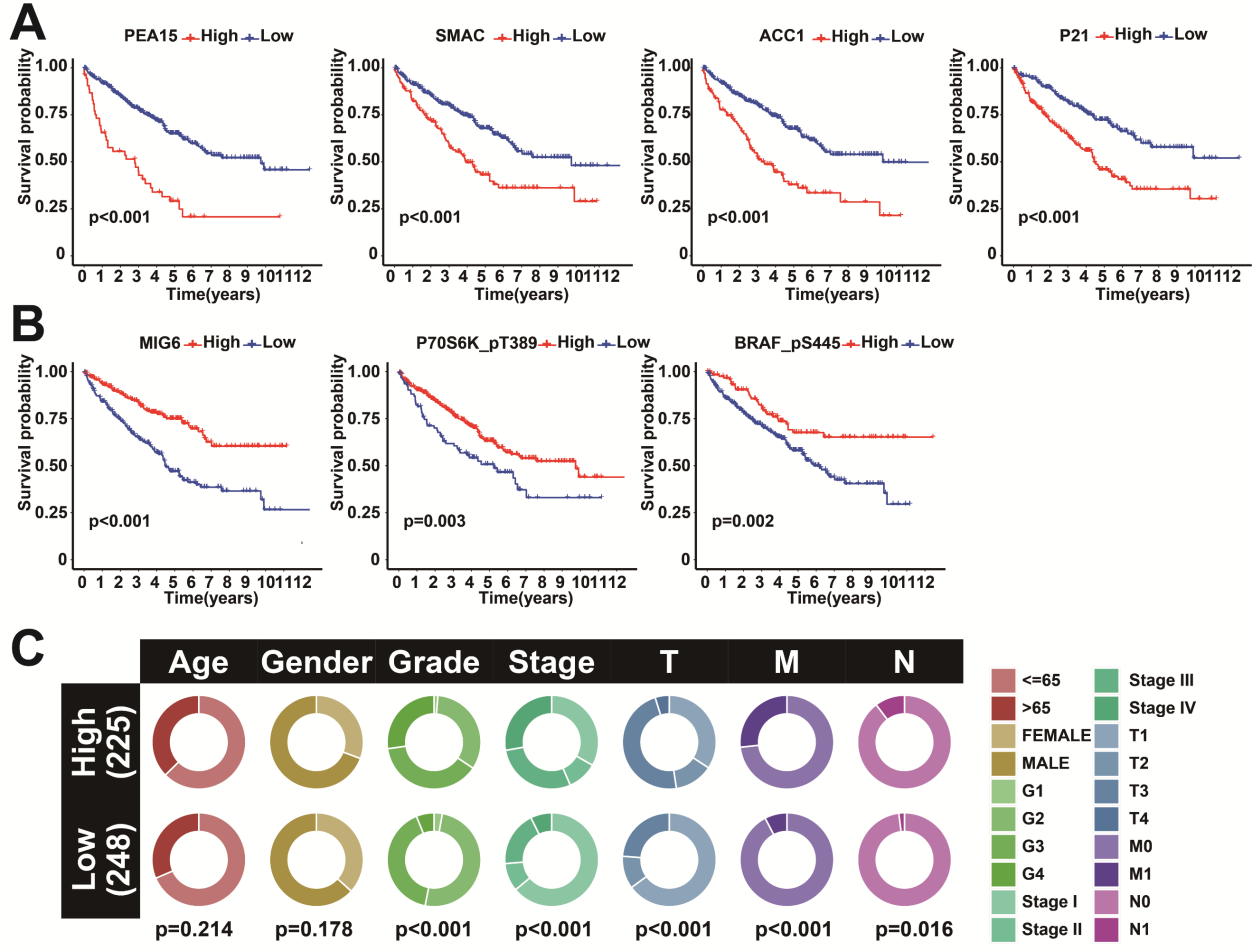
Survival analysis and interaction analysis of seven prognosis-related proteins. A: K-M survival curves between SMAC, P21, PEA15, and ACC1 expression and OS. B: K-M survival curves between MIG6, P70S6K_pT389, and BRAF_pS445 expression and OS. C: Correlation between the two risk score groups and clinical factors.

To further investigate the correlation between the 7 model proteins and the prognosis of KIRC, survival analyses were further performed. The results showed that high expression of SMAC, P21, PEA15, and ACC1 showed a more inferior prognosis (Figure 5B). In contrast, high expression of MIG6, P70S6K_pT389, and BRAF_pS445 showed a favorable prognosis (Figure 5A). This further demonstrated the feasibility of these seven proteins as prognostic markers. Clinical correlation analysis between the two risk subgroups showed that Grade, Stage, T, and M were significantly different between the two subgroups (Figure 5C). Co-expression analysis of the 7 predictive proteins revealed a total of 39 proteins that were significantly co-expressed (*r*>0.5 and *p*<0.001) (Figure S2A). The correlation analysis of the 7 prognostic proteins is visualized in the ring diagram (Figure S2B).

### Construction of nomogram

**Figure 6:**
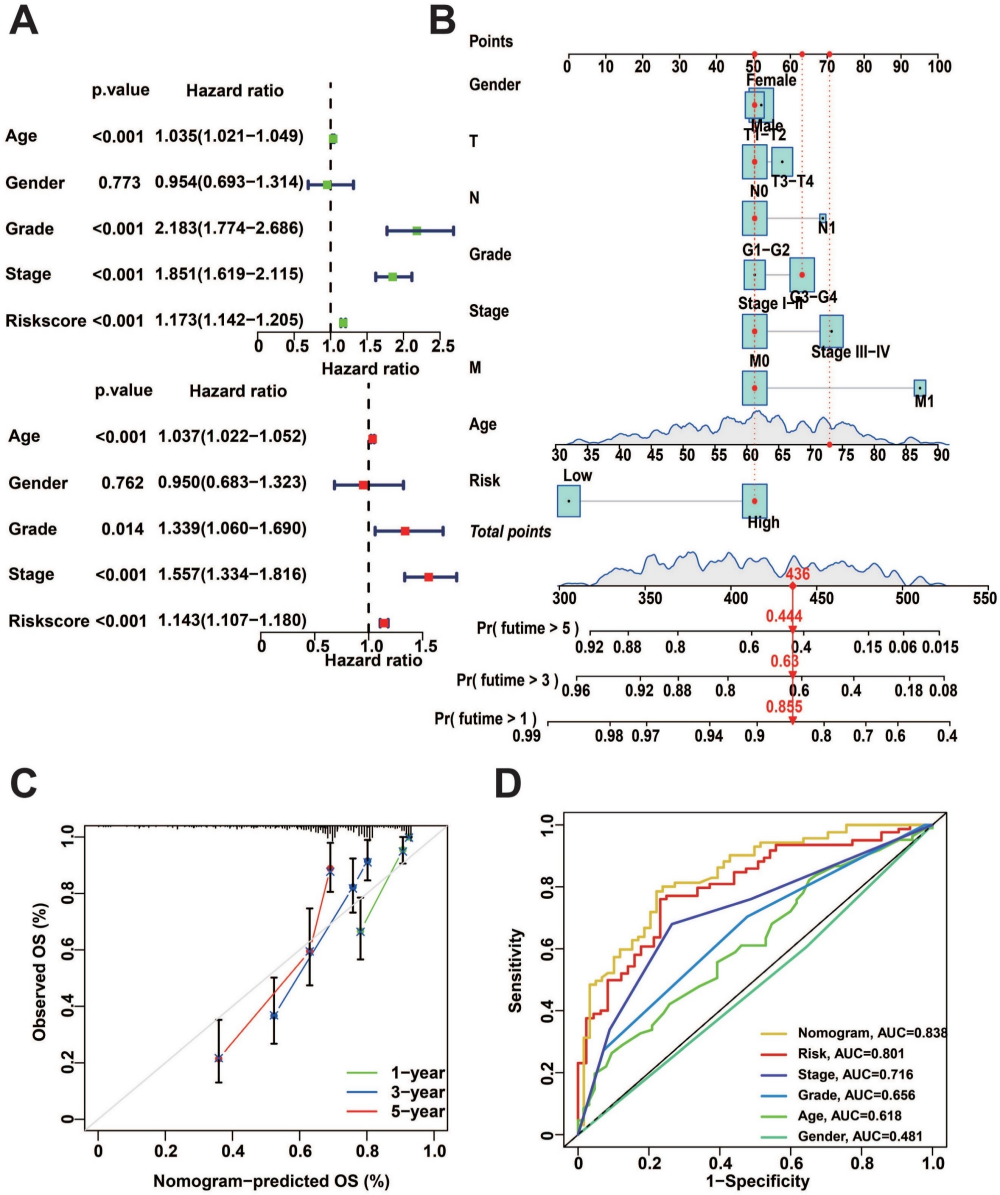
Nomogram construction and performance tests. A: Uni-variate and multi-variate COX regression analysis of clinical factors and risk score. B: Nomogram for predicting patient survival. C: Calibration curves for nomogram. D: Nomogram of ROC curves for OS prediction.

To better predict the prognosis of patients with KIRC, we performed uni- and multivariate Cox regression analysis, and the results showed that risk score, Age, Grade, and Stage were significantly correlated with the prognosis (Figure 6A). Subsequently, we utilized these clinical characteristics and risk scores to construct a nomogram. Calculating the total score from the scores of each independent factor in the nomogram could accurately predict the survival of patients (Figure 6B). The nomogram exhibits a promising predictive performance, as indicated by the calibration curves and ROC curves (Figure 6C, D).

### Evaluation of prognostically relevant protein subgroups

**Figure 7:**
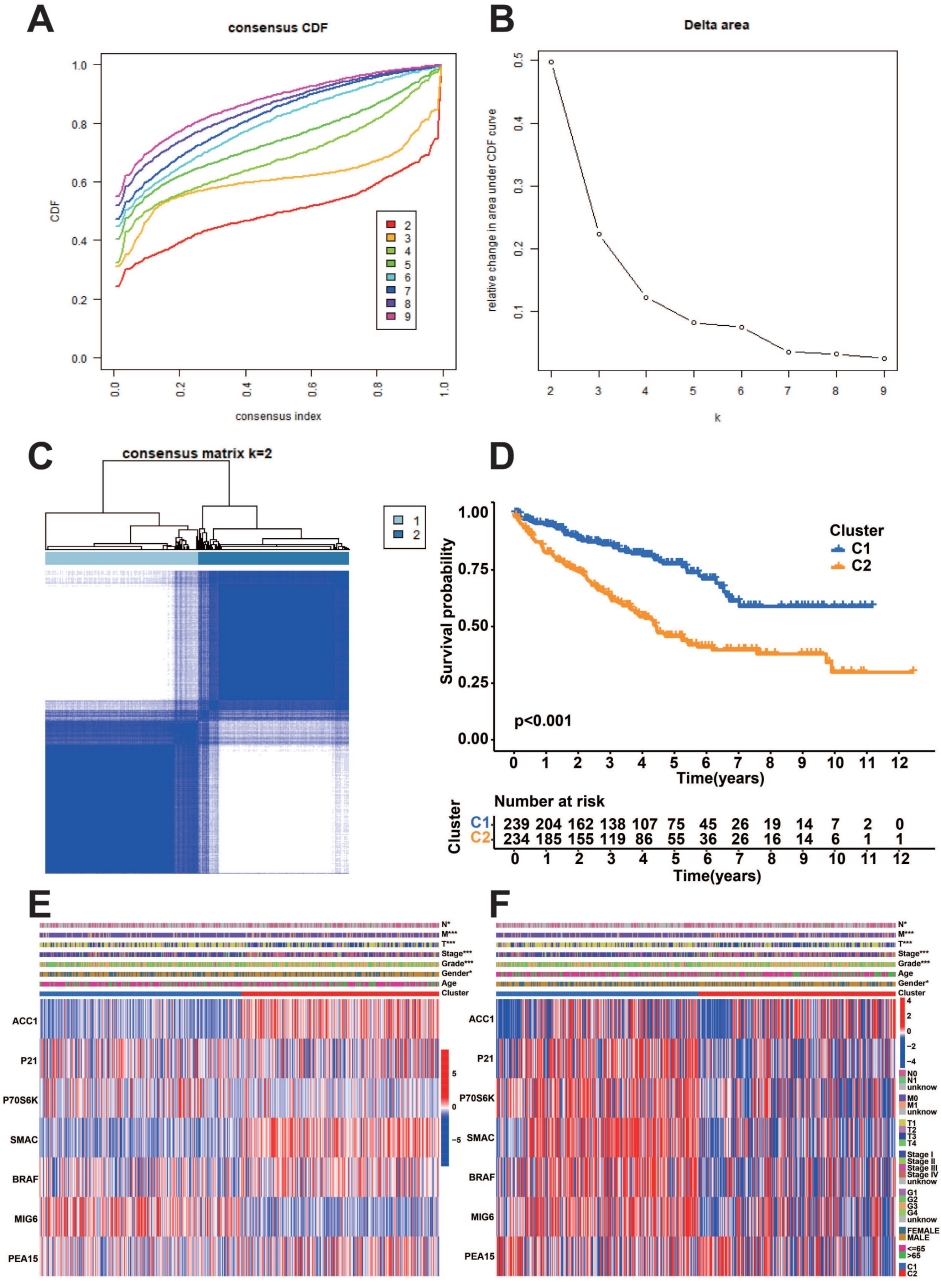
Consistency clustering analysis of prognostic proteins. A: Consistency clustering analysis cumulative distribution function (CDF) curve. B: K-means algorithm. C: Unsupervised clustering of seven model proteins and optimal consensus matrix with k = 2. D: Survival analysis of two prognostic protein subgroups. E-F: Heatmaps depicting the expression patterns of seven model proteins and protein-coding genes in two prognostic protein subgroups.

We performed a consistent clustering analysis based on the expression of model proteins to further identify the subgroups of model proteins. We found that the intra-group correlation was the most obvious and the inter-group correlation was lower when k=2 (Figure 7A-C). Afterward, an analysis of survival was conducted on the two subgroups of prognostic proteins. The results showed that the C1 group had a considerably more favorable prognosis (Figure 7D). Heatmaps illustrate the expression of model proteins and protein-coding genes across various subgroups. (Figure 7E-F).

### Immune cell infiltration and immune checkpoint analysis

**Figure 8:**
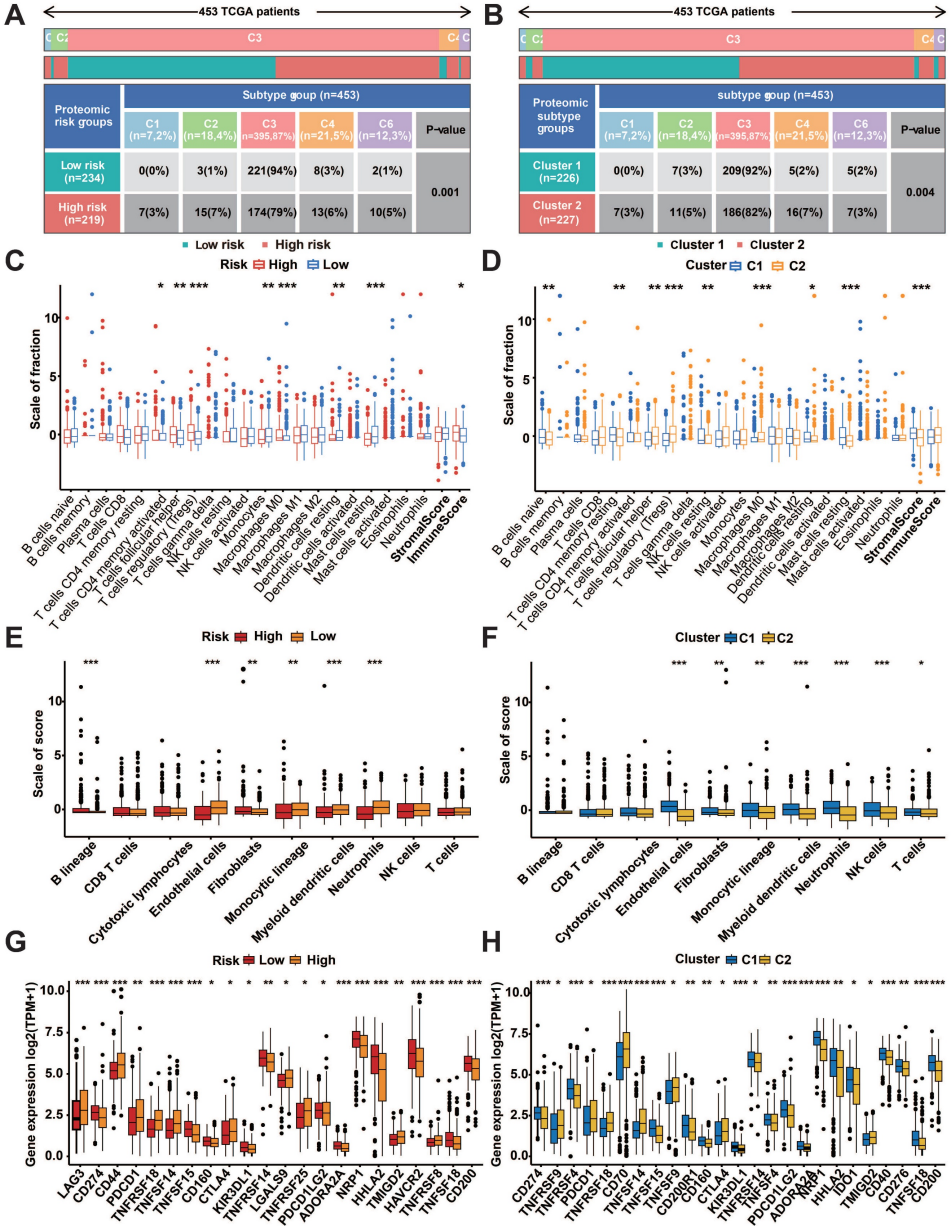
Immune infiltrates and subtypes of prognostic protein subgroups. A: Distribution of immune subtypes in risk subgroups. B: Immunosubtype distribution of prognostic protein subgroups C1 and C2. C: Divergences in the ratio of CIBERSORT immune cell infiltrations and TME scores among risk subgroups. D: Differences in proportions of CIBERSORT immune cell infiltration and TME scores for prognostic protein C1 and C2 subgroups. E: Divergences in the ratio of MCPcounter immune cell penetration among risk subgroups. F: The difference in the proportion of MCPcounter immune cell infiltration between prognostic protein C1 and C2 subgroups. G: Variations in the expression of immune checkpoint molecules among risk subgroups. H: Differences in immune checkpoint expression between prognostic protein C1 and C2 subgroups. (*p<0.05, **p<0.01, ***p<0.001, ****p<0.0001).

The examination of the distribution of immune subtypes in prognostic protein groupings, which were categorized into six subtypes in previous studies, revealed that the high-risk group had a higher prevalence of C1 (healing of wounds), C2 (dominated by IFN-g), C4 (depleted of lymphocytes), and C6 (dominated by TGF-b). Additionally, the low-risk group had a higher percentage of C3 (inflammatory) (Fig. 8A). Similar differences were found between prognostic protein C1 and C2 subgroups (Figure 8B). Immune infiltration analysis of transcriptomic data using CIBERSORT and MCPcounter showed significant differences in both risk subgroups and protein subgroups (*p*<0.05, Figure 7C-F). According to the tumor micro-environment (TME) analysis, the high-risk group exhibited considerably elevated immune scores (p < 0.05, Figure 8C). Additionally, the stromal scores of the C1 subgroup were significantly higher than those of the C2 subgroup (p<0.001, Figure 8D). TIDE analysis showed a high percentage of immunotherapy nonresponse in high-risk patients and the C2 subgroup (Figure S1H).

**Figure 9:**
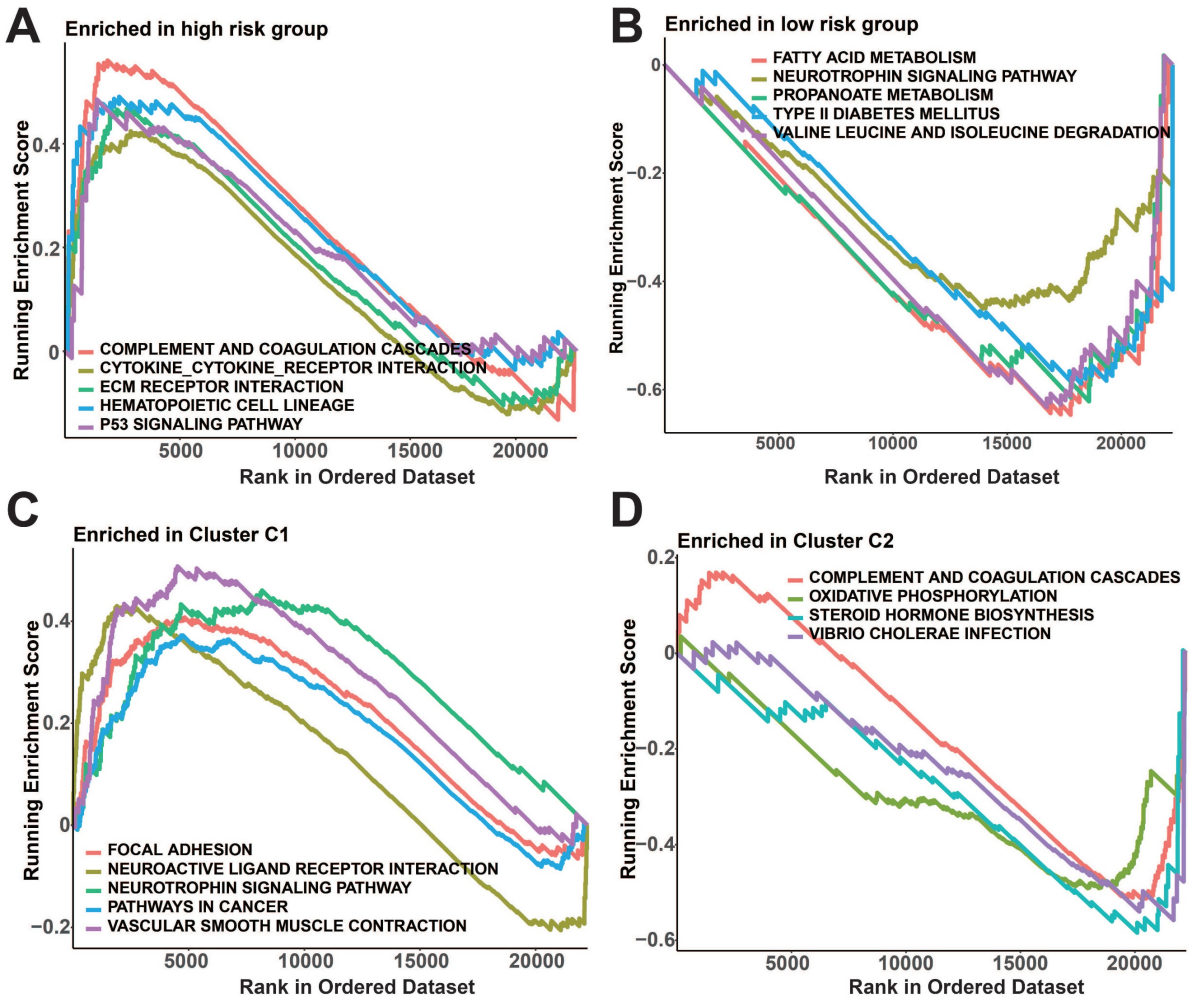
GSEA analysis of prognostic protein groupings. A-B: GSEA analysis of risk subgroups. C-D: GSEA analysis of protein subgroups.

Meanwhile, notable variations in immune checkpoint manifestation were observed among the two risk groups as well as the protein subgroups (*p*<0.05, Figure 8G, H). In the high-risk group, GSEA revealed enrichment of complement and coagulation cascades, cytokine receptor interaction, ECM receptor interaction, hematopoietic cell lineage, and p53 signaling pathway (Figure 9A). In addition, fatty acid metabolism, neurotrophic protein signaling pathways, propionic acid metabolism, type II diabetes, and valine, leucine, and isoleucine degradation pathways were enriched in the low-risk group (Figure 9B). Figure 9C shows that the prognostic protein C1 subgroup had enriched pathways including focal adhesion, interaction of neuroactive ligands and receptors, neurotrophin signaling pathway, pathways related to cancer, and contraction of vascular smooth muscle. In Figure 9D, the complement and coagulation cascades, oxidative phosphorylation, steroid hormone biosynthesis, and vibrio cholerae infection pathways were found to be more abundant in the C2 subgroup.

### The relationship between the expression of prognostic protein-coding genes and prognosis

**Figure 10:**
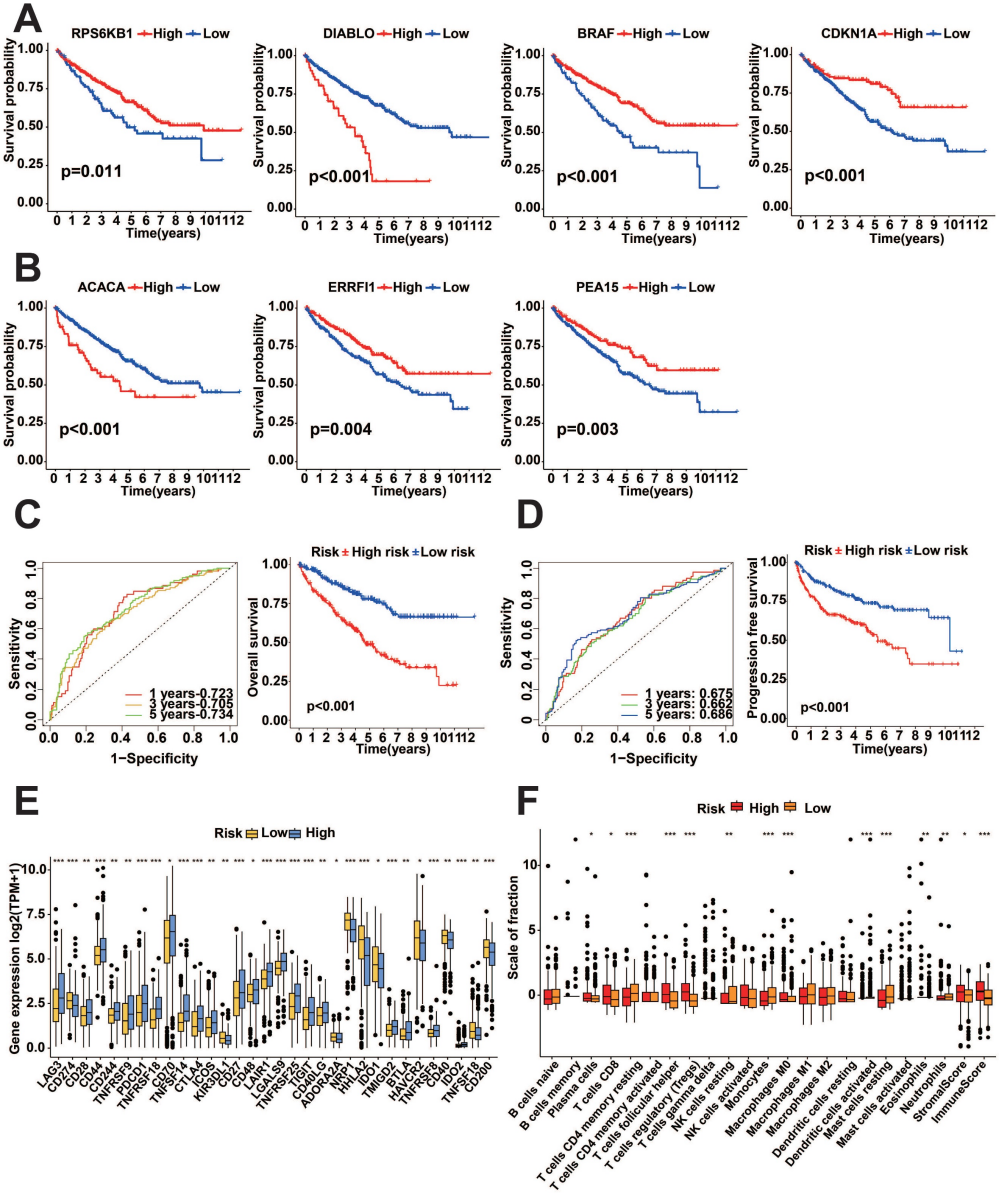
Survival analysis of TCGA-KIRC prognostic protein-encoding genes. A-B: K-M survival curves of prognostic protein-encoding genes for survival analysis. C: K-M survival curves and ROC curves for OS survival analysis for protein-coding genes. D: K-M survival curves and ROC curves for PFI survival analysis for protein-coding genes. E: Differential evaluation of immune checkpoint expression for risk subgroups of protein-coding genes. F: Analysis of immune infiltration in risk subgroups of protein-coding genes. (*p<0.05, **p<0.01, ***p<0.001, ****p<0.0001).

We performed a survival analysis of these seven genes, which showed that all seven genes were significantly associated with prognosis, with high expression of DIABLO (encodes the SMAC protein) and ACACA (encodes the ACC1 protein) showing worse prognosis, while low expression of PEA15 (encodes the PEA15 protein), CDKN1A (encodes P21 protein), ERRFI1 (which encodes MIG6 protein), RPS6KB1 (encodes the P70S6K_pT389 protein) and BRAF (encodes the BRAF_pS445 protein), showed worse prognosis (*p*<0.05, Figure 10A-B). To examine the predictive effect of the seven protein-coding genes on prognosis, we constructed the Cox proportional hazards model using these seven genes. The survival analysis indicated that the model built using these seven genes exhibited outstanding predictive ability. K-M survival analysis showed worse prognostic performance in the high-risk score group (*p*<0.05, Figure 10C-D). Analysis of 47 immune checkpoints revealed that 32 of these immune checkpoint genes exhibited distinct expression patterns (*p*<0.05, Figure 10E). The analysis of ESTIMATE showed that the immune score and stromal score were higher in the high-risk group (*p*<0.05, Figure 10F).

### Analysis of prognostic protein-coding genes using online databases

**Figure 11:**
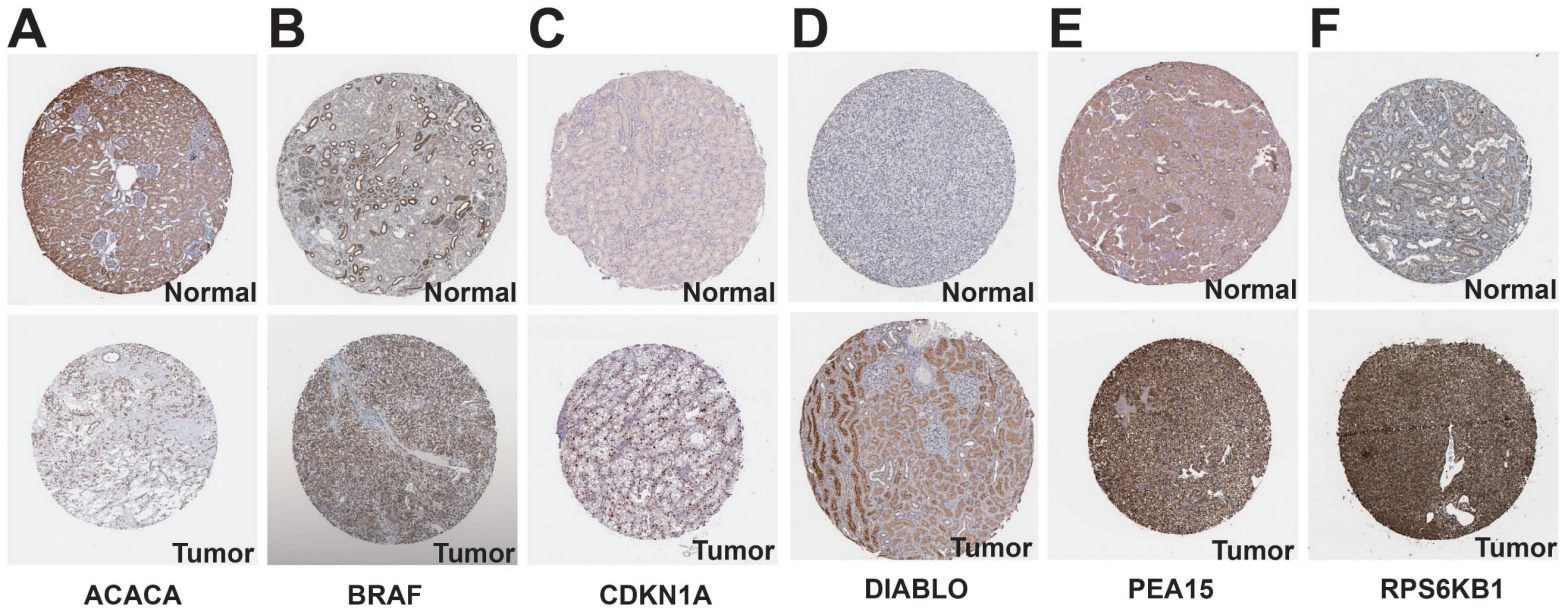
Expression of prognostic protein-encoding genes in tumor tissues and normal tissues in the HPA database. A: ACACA. B: BRAF. C: CDKN1A. D: DIABLO. E: PEA15. F: RPS6KB1.

**Figure 12:**
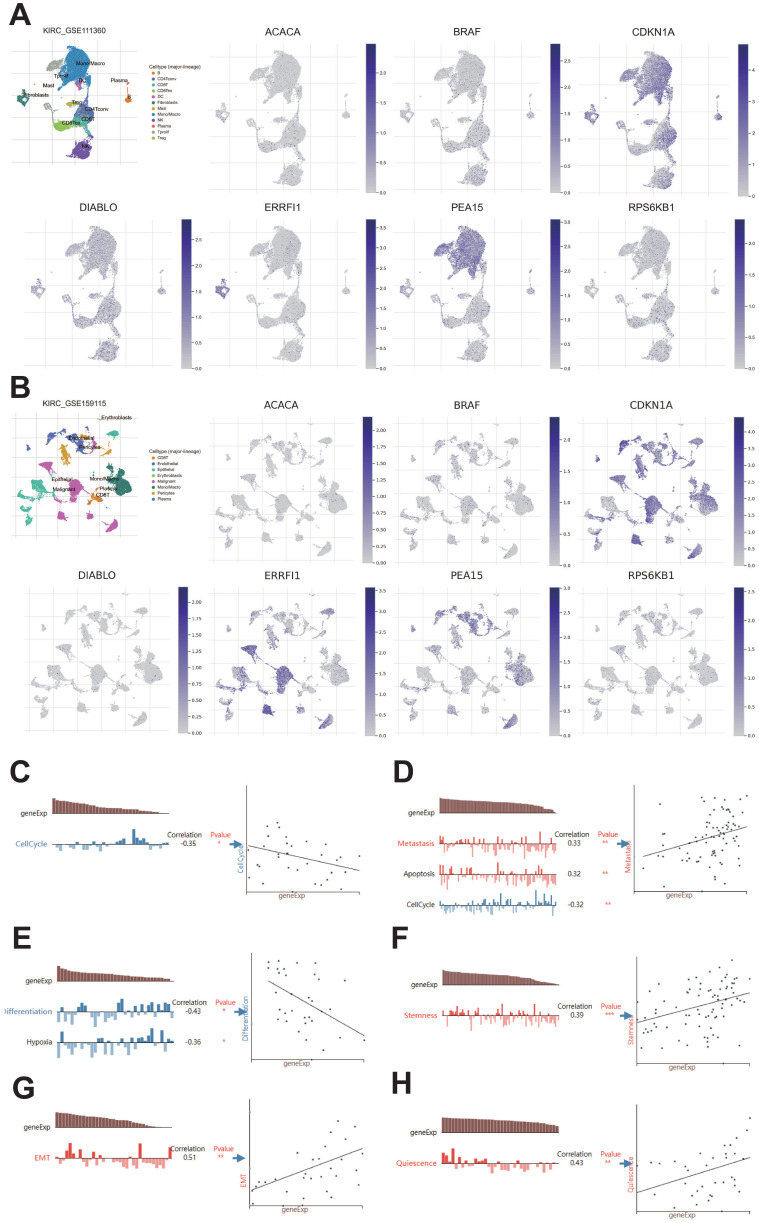
TISCH database and CancerSEA database analysis of protein-coding genes. A: Gene expression from the KIRC-GSE111360 dataset in the TISCH database. B: Gene expression from the KIRC-GSE159115 dataset in the TISCH database. C-H: Correlation of prognostic protein-encoding genes with cancer cell functional status at the single-cell level.

We examined the HPA database to analyze the expression of protein-coding genes. The results indicated that ACACA exhibited low expression in KIRC tissues, while BRAF, CDKN1A, DIABLO, PEA15, and RPS6KB1 showed high expression in KIRC tissues (Figure 11). To examine the variations in gene expression across various cell profiles, we utilized the TISCH database's KIRC_GSE111360 and KIRC_GSE159115 datasets to detect the expression levels of protein-coding genes (Figure 12A-B). The correlation between protein-coding genes and the functional status of tumor cells was analyzed using the CancerSEA database. The results revealed that ACACA was negatively correlated with the cell cycle (*r*=-0.35, *p*<0.05, Figure 12C), CDKN1A was positively associated with metastasis (*r*=0.33, *p*<0.01, Figure 12D), RPS6KB1 was negatively related to invasion (*r*=-0.38, *p*<0.001, Figure 12E), BRAF was positively linked to Stemness (*r* =0.39,* p*<0.001, Figure 12F), ERRFI1 was positively connected to epithelial-mesenchymal transition (EMT) (*r*=0.51, *p*<0.01, Figure 12G), and PEA15 was positively correlated with Quiescence (*r*=0.43, *p*< 0.01, Figure 12H).

### Verification of protein-coding genes by qRT-PCR

**Figure 13:**
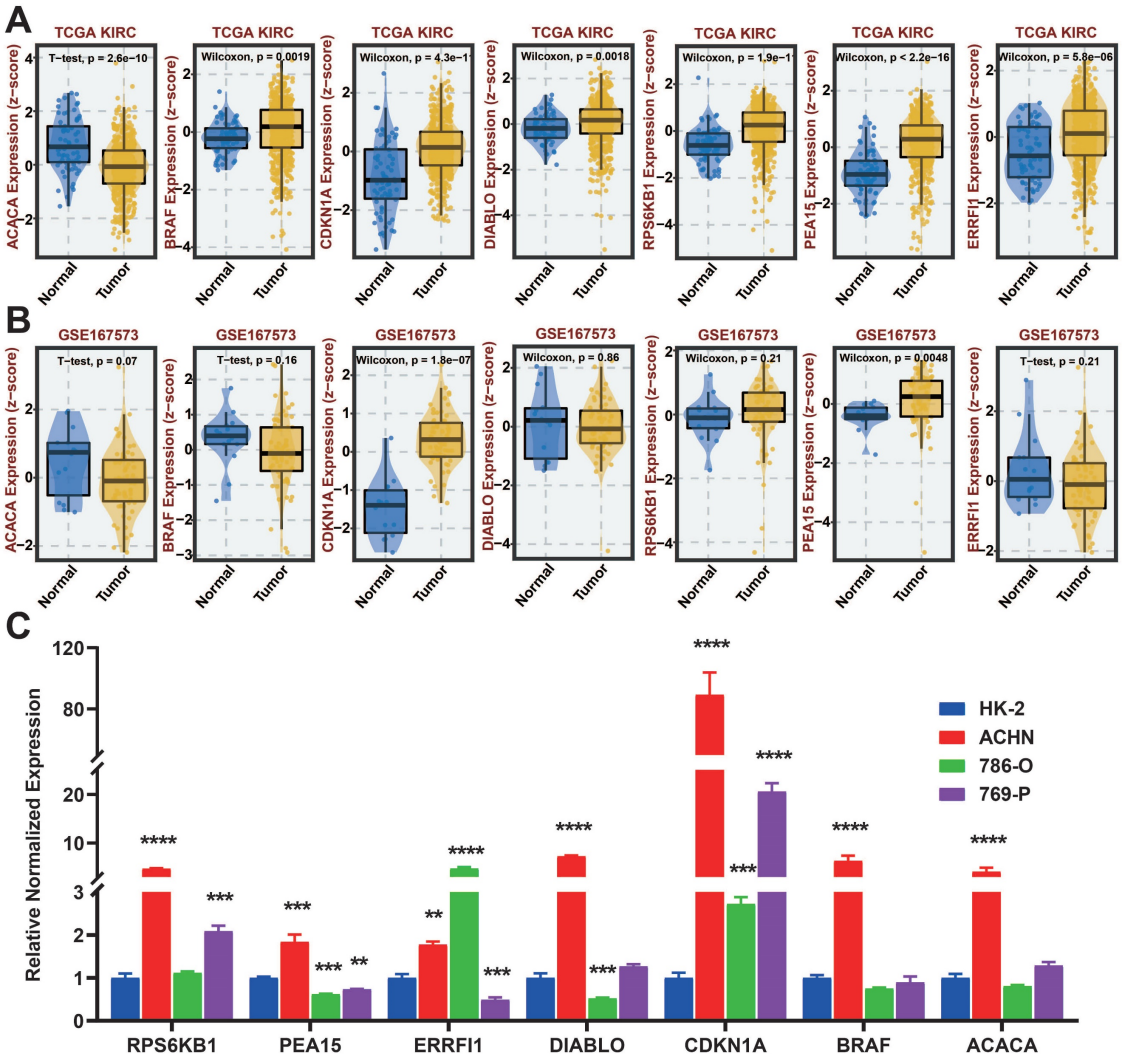
qRT-PCR of seven protein-coding genes. A: Gene expression from the TCGA-KIRC dataset in the BEST website. B: Gene expression from the GSE167573 dataset in the BEST website. C: Expression of seven protein-coding genes in cell lines examined by qRT-PCR.

We analyzed the differences in expression of seven protein-coding genes between tumor and normal samples at the BEST website (Figure 13A-B). To further investigate the manifestation of these seven protein-encoding genes in cellular models, we analyzed their presence in four different cell lines, namely HK-2, ACHN, 786-O, and 769-P, through the utilization of qRT-PCR. The results show that BRAF, CDKN1A, DIABLO, PEA15, ERRFI1, and RPS6KB1 are highly expressed in tumor cell lines, which is the same as in the TCGA database. Nevertheless, the cellular expression of ACACA varied from that observed in TCGA, potentially due to the disparity in sample size (Figure 13C).

## Discussion

KIRC has an insidious onset, is heterogeneous, and is insensitive to conventional radiotherapy^12^, ^13^. Partial or radical nephrectomy is the primary approach for treating non-metastatic KIRC. After undergoing surgical treatment, around 25%~50% of patients still experience either local recurrence or distant metastasis^14^-^16^. At present, focused immunotherapies are employed for advanced renal cancer that has spread to distant areas^17^, although the effectiveness of these treatments for KIRC patients remains restricted^18^. In this particular situation, healthcare professionals require more accurate biological indicators to establish risk categorization for anticipating patient prognosis and response to immunotherapy, as well as to offer direction for clinical diagnosis and treatment.

By conducting survival analysis and LASSO on the training cohort, we developed a prognostic model that consists of seven proteins. The model's predictive performance was validated in the test cohort, showcasing its exceptional accuracy. Furthermore, the nomogram was developed to forecast survival by amalgamating the risk scores of the prognostic proteins with clinical factors including age, gender, tumor stage, and grading.

Earlier studies on the proteomic characterization of KIRC have demonstrated a strong association between proteomics and the tumor immune microenvironment. These studies have also revealed that distinct immune subtypes exhibit diverse clinical characteristics, prognoses, and responses to treatment^10^. Hence, we conducted analyses on immune cell infiltration and immune checkpoint expression in various risk groups and subtype subgroups, revealing noteworthy disparities among the different subgroups. Analysis of prognostic protein subtypes also indicated that the C1 cluster exhibited a significantly more favorable prognosis compared to the C2 cluster. There are also studies showing that the Pearson correlation between protein and mRNA data was higher than 0.75 in immune and stromal-derived features^19^. Hence, we additionally conducted a survival assessment on predictive protein-coding genes and developed a reliable prognostic model using them, demonstrating strong predictive accuracy.

There are often intrinsic mechanisms underlying the prognostic differences between risk groups^20^, and our GSEA results showed significant enrichment of complement and coagulation cascades, cytokine receptor interactions, ECM receptor interactions, hematopoietic cell lineage, and p53 signaling pathway in the high-risk group. Activation of the complement system and coagulation cascade in the tumor microenvironment promotes inflammatory response and tumor progression^21^, ^22^. Cytokine receptor interactions regulate immune responses and inflammatory processes^23^, while ECM receptor interactions play a key role in tumor growth and metastasis^24^, and finally, p53 is an important tumor suppressor gene^25^, which suggests that dysfunction of p53 may be present in the high-risk group, and that the above results may be associated with a poorer prognosis in the high-risk group. In contrast, the low-risk group was significantly enriched in metabolism-related signaling pathways, suggesting that the metabolic status of patients in the low-risk group may be associated with their better prognosis^26^. We suggest further studies to determine whether these pathways may serve as new therapeutic targets or biomarkers.

Among the prognostic protein-coding genes, ACACA played a crucial role in lipid metabolism as a significant enzyme and as a key enzyme in regulating the initial production of fatty acids 27. Bioactive lipids could promote or inhibit the development and metastasis of renal cancer by regulating the stability and transcriptional activity of HIF-2α, thus affecting the proliferation, migration, angiogenesis, immune escape, and other processes of renal cancer cells^28^, ^29^. It has been shown that ACACA-induced lipid synthesis and lipid accumulation led to hepatocellular carcinoma progression^30^, whereas inhibition of ACACA similarly inhibited hepatocellular carcinoma progression^31^. The research focused on ACACA and kidney cancer has not made significant advancements. However, compounds that inhibit lipid absorption or transportation have demonstrated anti-cancer effects in laboratory and animal studies. Furthermore, a number of these medications have progressed to clinical trials 32. The p21 protein, encoded by the gene CDKN1A, interacted with a variety of proteins involved in cell cycle inhibition, as well as cell differentiation, and migration, thereby acting as an oncogenic or pro-oncogenic agent^33^, ^34^. Up-regulation of P21 signaling or p21 mRNA stability was found to inhibit the growth of KIRC 35, 36. Immunohistochemical analysis of tissue microarrays from 366 KIRC patients showed that p21 might contribute to prognosis and play different biological functions in limited and metastatic renal cell carcinoma^37^. The RPS6KB1 gene encodes ribosomal protein S6 kinase (p70S6K), which is used to control cell survival, proliferation, and metabolism through the PI3K/mTOR signaling pathway^38^. Several studies have shown that p70S6K is associated with the pathophysiology of various tumors such as prostate and colorectal cancers^39^-^41^. Smac was a pro-apoptotic mitochondrial protein, and phospholipid metabolism and phosphatidylethanolamine synthesis regulated by Smac and phosphatidylethanolamine interactions were critical for cancer cell proliferation^42^, ^43^. Studies have demonstrated that patients with RCC who exhibited low levels of Smac expression had a fourfold increase in the risk of death compared to patients with high expression^44^.

BRAF functioned as a serine/threonine kinase that played a role in controlling the MAPK signaling pathway. BRAF was mutated in a variety of cancers, leading to aberrant activation of the signaling pathway. Currently, the FDA has approved small molecule inhibitors that target BRAF for the treatment of advanced melanoma and non-small cell lung cancer^45^, ^46^. ERRFI1 acts as a suppressor of EGFR by directly attaching to EGFR, restraining the enzymatic function of EGFR, and facilitating the degradation of EGFR in lysosomes^47^. Research has indicated that ERRFI1 is significant in the development of lung cancer, endometrial cancer, and breast cancer^48^. PEA15 was widely expressed in breast cancer, and studies have shown that PEA15 dephosphorylation might be associated with breast cancer progression and drug resistance^49^, ^50^.

Although our model exhibits excellent predictive performance, there are still certain constraints in our research. Our study was based on the analysis and validation of data collected through public databases, the inclusion of corresponding external validation data may give better results, and prospective studies are needed to further evaluate the model. Moreover, it is crucial to conduct thorough functional experiments to clarify the interrelated mechanisms of the seven prognostic proteins.

## Conclusion

In general, we created a proteomics-driven framework that can forecast the outlook, immune surroundings, and medication reactions of patients with KIRC. The model relies on 7 prognostic proteins and clinical factors. The model's potential for reliable prognostic prediction in KIRC was demonstrated through validation against the HPA database and qRT-PCR experiments.

## Supplementary Material

Supplementary figures and table.

## Figures and Tables

**Table 1:** Primers for qRT-PCR.

Gene	Forward Primer Sequence (5' to 3')	Reverse Primer Sequence (5' to 3')
ERRFI1	GGGCAGGGTATCCATTCT	TCCCTCAACAAGACGCA
RPS6KB1	ATATTTGCCATGAAGGTGCT	CGATGAAGGGATGCTTTACT
CDKN1A	CTGGCCCCTCAAATCGT	CCGCTGCTAATCAAAGTGC
ACACA	GAAGGGGTTTTCACTGTCC	GAGGATCGTATGGGGTCTT
BRAF	CAAATTCTCACCAGTCCGT	GGTCTCGTTGCCCAAAT
ACTB	TCTCCCAAGTCCACACAGG	GGCACGAAGGCTCATCA
PEA15	TGAGGAGGATGAGCTGGA	GGGAGTGGTCTGATGAAGG
DIABLO	CCTACCTGCGTGAGGATTG	GGATCTGCCGCCTCTTC

## Abbreviations

KIRC: Kidney clear cell carcinoma
TCGA: The Cancer Genome Atlas Program
qRT-PCR: Quantitative Real-time PCR
OS: Overall survival
PFI: Progression Free Interval
RCC: Renal cell carcinoma
PFS: Progression-free survival
GDC: Genomic Data Commons
LASSO: Least absolute shrinkage and selection operator
ROC: Receiver Operating Characteristic
GSEA: Gene set enrichment analysis
TIDE: Tumor Immune Dysfunction and Exclusion
HPA: Human Protein Atlas
TISCH: Tumor Immune Single-cell Hub
CancerSEA: Cancer Single-cell State Atlas
FBS: Fetal bovine serum
P/S: Penicillin-streptomycin
K-M: Kaplan-Meier
PCA: Principal component analysis
CNV: Copy number variation
TMB: Tumor mutational load
CDF: Cumulative Distribution Function
TME: Tumor micro-environment
EMT: Epithelial-Mesenchymal Transition
