# Supplementary material for: Integration of proteomics and transcriptomics to construct a prognostic signature of renal clear cell carcinoma
Source: Int J Med Sci. 2024 Aug 19;21(11):2215–32. doi: 10.7150/ijms.99992 (PMC11373548; doi:10.7150/ijms.99992)
Supplement: Supplementary file 1 — Supplementary figures and table. [file ijmsv21p2215s1.zip › Supplementary Figure S2.pdf]

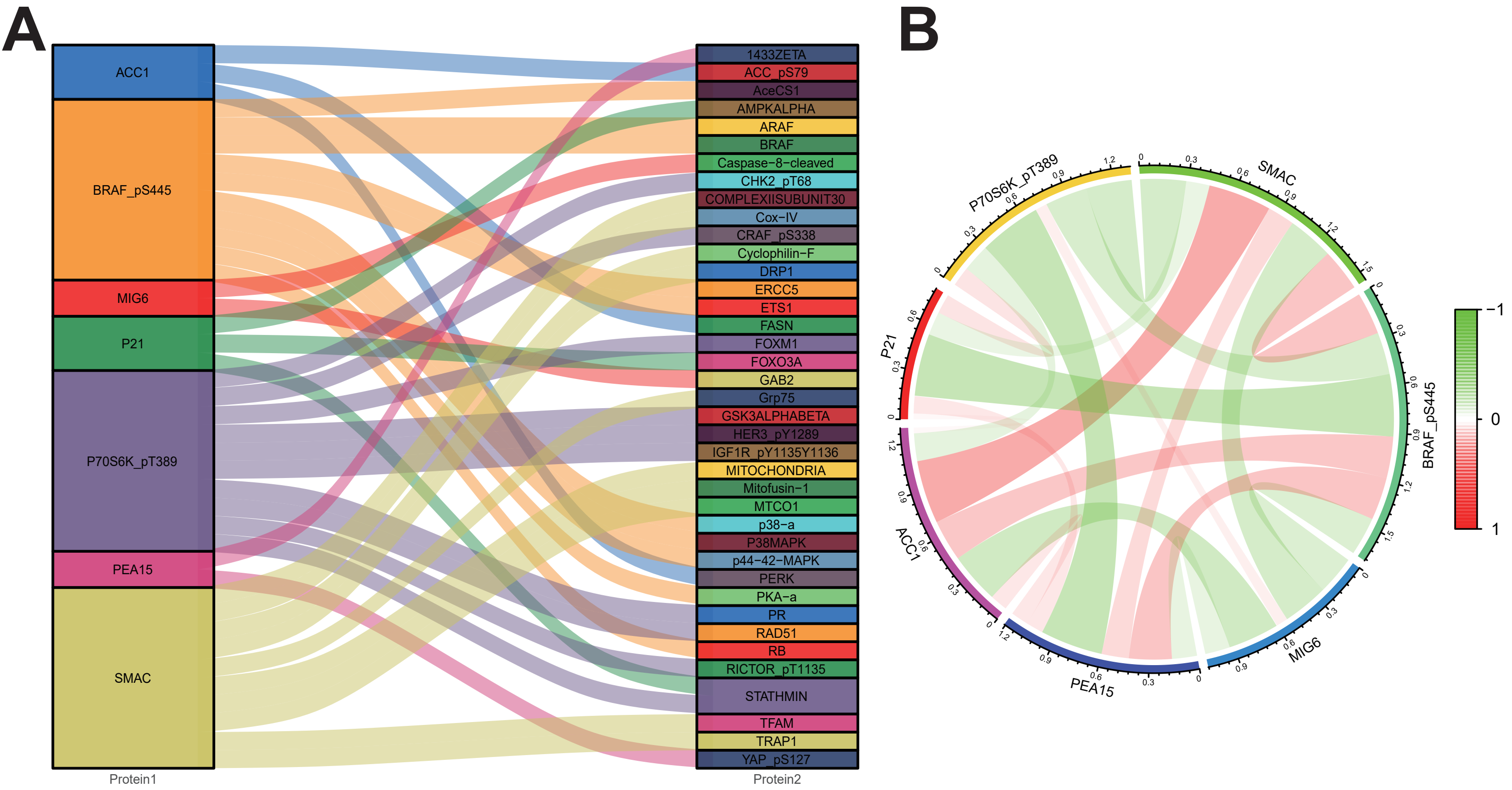


**Figure S2.** (A) Co-expression Sankey diagram of the 7 model proteins. (B) Interrelationships among the 7 model proteins.
