# Supplementary material for: Integration of proteomics and transcriptomics to construct a prognostic signature of renal clear cell carcinoma
Source: Int J Med Sci. 2024 Aug 19;21(11):2215–32. doi: 10.7150/ijms.99992 (PMC11373548; doi:10.7150/ijms.99992)
Supplement: Supplementary file 1 — Supplementary figures and table. [file Supplementary Figure S1.pdf › Supplementary Figure S1.pdf]

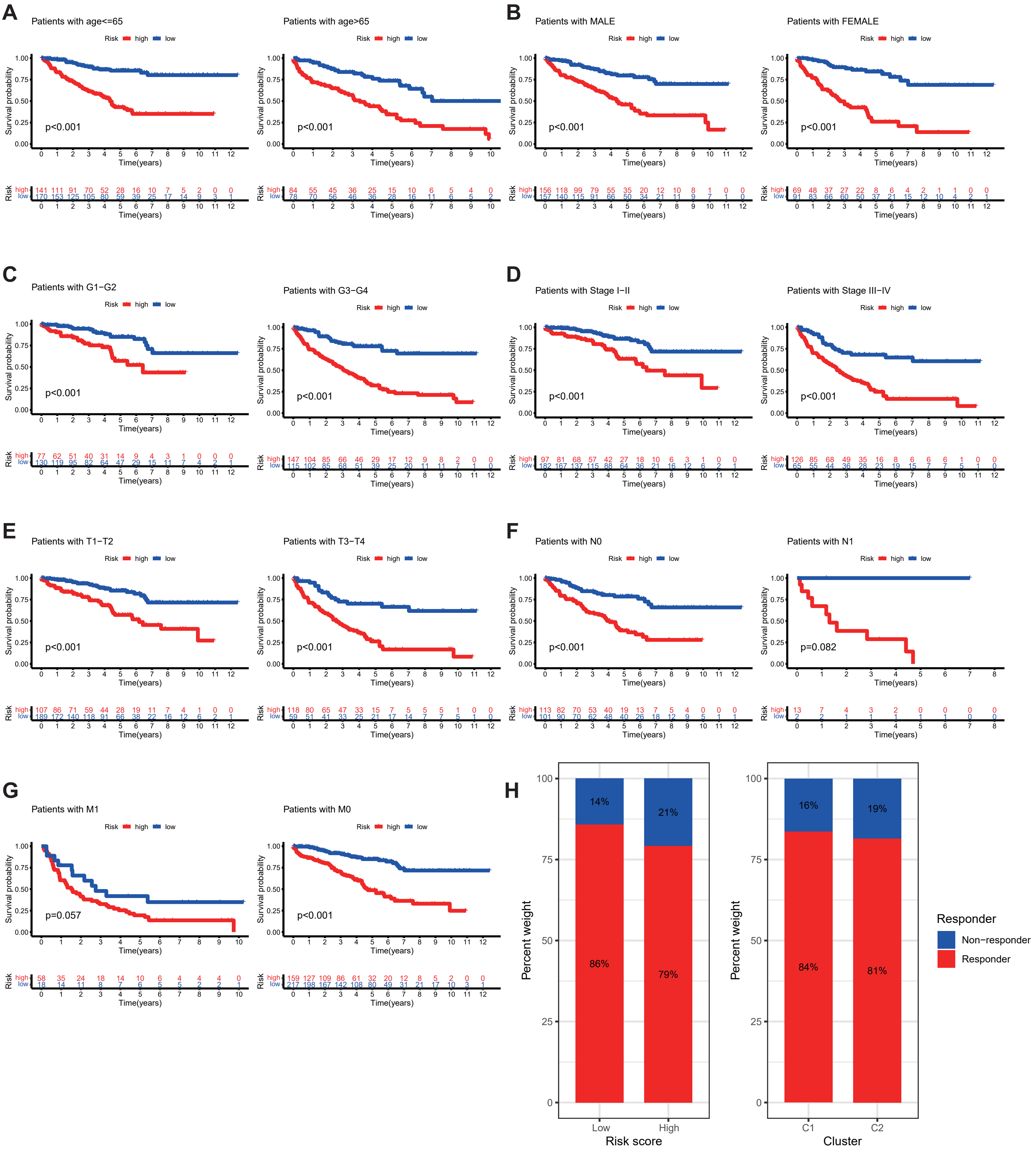

Figure S1. Kaplan-Meier survival analysis of samples with different clinical factors in different risk subgroups and the percentage of immunotherapy response in different subgroups. (A) Kaplan-Meier survival analysis of overall survival (OS) in different age subgroups. (B) Kaplan-Meier survival analysis of OS in different gender subgroups. (C) Kaplan-Meier survival analysis of OS in different graded subgroups. (D) Kaplan-Meier survival analysis of OS for different clinical stages. (E) Kaplan-Meier survival analysis of OS for different primary tumor size stages. (F) Kaplan-Meier survival analysis of OS with different lymph node infiltration grades. (G) Kaplan-Meier survival analysis of OS with different metastatic staging.(H) Differences in immunotherapy response across risk groups and subgroups.
